# An integrated systems biology and machine learning framework for identifying potential biomarkers and pathways in autism spectrum disorder

**DOI:** 10.1371/journal.pone.0355984

**Published:** 2026-08-24

**Authors:** Sara Hosseinpoor, Hakimeh Zali, Hassan Zohrevand, Seyed Amir Mirmotalebisohi, Fariba Khodagholi, Maryam Bazrgar, Sareh Asadi, Abolhassan Ahmadiani

**Affiliations:** 1 Neuroscience Research Center, Institute of Neuroscience and Cognition, Shahid Beheshti University of Medical Sciences, Tehran, Iran; 2 Department of Tissue Engineering and Applied Cell Sciences, School of Advanced Technologies in Medicine, Shahid Beheshti University of Medical Sciences, Tehran, Iran; 3 Student Research Committee, Department of Biomedical Engineering and Medical Physics, School of Medicine, Shahid Beheshti University of Medical Science, Tehran, Iran; 4 Student Research Committee, School of Advanced Technologies in Medicine, Shahid Beheshti University of Medical Sciences, Tehran, Iran; 5 Cellular and Molecular Biology Research Center, Shahid Beheshti University of Medical Sciences, Tehran, Iran; 6 Neurobiology Research Center, Institute of Neuroscience and Cognition, Shahid Beheshti University of Medical Sciences, Tehran, Iran; Amity University Noida, INDIA

## Abstract

**Background:**

Autism spectrum disorders (ASD) are a group of neurodevelopmental disorders whose underlying molecular mechanisms and biological processes remain incompletely understood. In this study, we used a multi-layered systems biology approach to prioritize candidate genes and regulatory factors associated with ASD.

**Method:**

Gene expression data from peripheral blood samples were obtained from the Gene Expression Omnibus (GEO) database (GSE18123). Using analyses performed in R software, differentially expressed genes (DEGs) in patients with ASD were identified (p-value < 0.05 and |log_2_FC| > 0.5). These DEGs were used to perform weighted gene co-expression network analysis (WGCNA) and construct a protein–protein interaction (PPI) network. By integrating the results of these network analyses with feature selection techniques (LASSO and random forest feature importance), candidate genes associated with ASD were prioritized and evaluated using qRT-PCR in the valproic acid (VPA)-induced rat model of autism. Furthermore, a gene regulatory network (GRN) was constructed to identify the regulatory factors associated with DEGs.

**Result:**

*TLR8* and *CASP4* were prioritized as candidate genes that may be associated with ASD, because they were located within the co-expression module that showed the strongest correlation with ASD, were identified as key nodes of the PPI network, and were selected by feature selection algorithms. Our experimental validation showed increased expression of *TLR8* and *CASP4* in the autism model compared with controls; *TLR8* was upregulated in both the hippocampus and peripheral blood, whereas *CASP4* was upregulated only in the hippocampus. Furthermore, GRN analysis identified miR-891b and miR-627-3p as potential regulators of *TLR8*, and miR-26b-5p as associated with *CASP4*.

**Conclusion:**

These findings indicate that *CASP4* and *TLR8*, together with their associated regulatory miRNAs, may represent promising biomarkers and potential therapeutic targets for future ASD research and contribute to a better understanding of the pathophysiological mechanisms underlying ASD.

## 1. Introduction

ASD is a neurodevelopmental disorder characterized by deficits in social communication, impaired interactions, and repetitive behaviors [1]. According to a meta-analysis, ASD is estimated to affect 0.77% of children worldwide [2]. Evidence from studies suggests that ASD is caused by complex interactions between genetic, epigenetic, and environmental factors that lead to changes in brain structure and function [3,4]. Recent studies have identified a large number of genes that are differentially expressed in various tissues, such as the brain, peripheral blood, and gastrointestinal, in individuals with ASD compared to controls [5]. Given the genetic heterogeneity of ASD, transcriptional data provide valuable insights into its underlying molecular mechanisms. For example, bioinformatics analyses of gene expression data indicate that ASD-associated genes converge on biological pathways, including synaptic function, immune responses, ion channel activity, and cell cycle regulation [6]. These findings suggest that identifying common molecular alterations, regardless of diverse genetic backgrounds, is essential for improving our understanding of the underlying pathophysiological mechanisms of ASD.

Gene expression changes can arise from disruptions in regulatory mechanisms. Transcription factors (TFs) and non-coding RNAs, particularly miRNAs, are key elements that regulate gene expression [7,8]. Therefore, their study is essential to understanding the mechanisms that lead to differential gene expression in ASD patients. In this context, Gosha et al. (2025) identified several TFs as potential regulators of key genes associated with ASD. For example, *VEZF1* and *FOXP2* were among the TFs identified in their study, which are associated with processes related to brain morphogenesis and development [9]. Studies comparing miRNA expression profiles in individuals with ASD and controls have also identified a significant number of differentially expressed miRNAs. Among them, miR-23a-3p, miR-27a-3p, miR-106b-5p, miR-93-5p, miR-7-5p, miR-146a-5p, and miR-155-5p have been consistently reported across multiple studies and are known to target a substantial number of risk genes associated with ASD [10]. Despite these promising findings, no reliable biomarkers or therapeutic targets have yet been established. Consequently, efforts to identify non-invasive and specific biomarkers, as well as effective therapeutic targets for individuals with ASD, remain ongoing.

Recent research highlights the value of a systems biology approach in revealing the molecular pathways disrupted in neurodevelopmental disorders [11] and other complex diseases [12,13]. Therefore, the present study aims to identify key molecular signatures of ASD using an integrative systems biology framework applied to peripheral blood gene expression profiles. By integrating network-based analyses and machine learning approaches, we seek to prioritize candidate genes and explore their potential regulatory factors. This integrative strategy not only enhances our understanding of ASD pathophysiology but also may support the discovery of novel biomarkers and therapeutic targets for these patients. In addition, we evaluate the expression levels of the identified candidate genes in the VPA-induced rat model of autism compared with control animals.

## 2. Materials and methods

### 2.1. Overview of study design

In this study, gene expression profiles from the peripheral blood of individuals with and without ASD were obtained from the GEO database and analyzed using R software to identify DEGs between ASD patients and healthy controls. These DEGs were then used to construct gene co-expression and PPI networks. Feature selection techniques were subsequently applied to prioritize genes most strongly associated with ASD. To explore the regulatory landscape, four types of interactions—miRNA-gene, TF-gene, miRNA-TF, and TF-miRNA—were retrieved, culminating in the construction of a comprehensive GRN.

**Fig 1:**
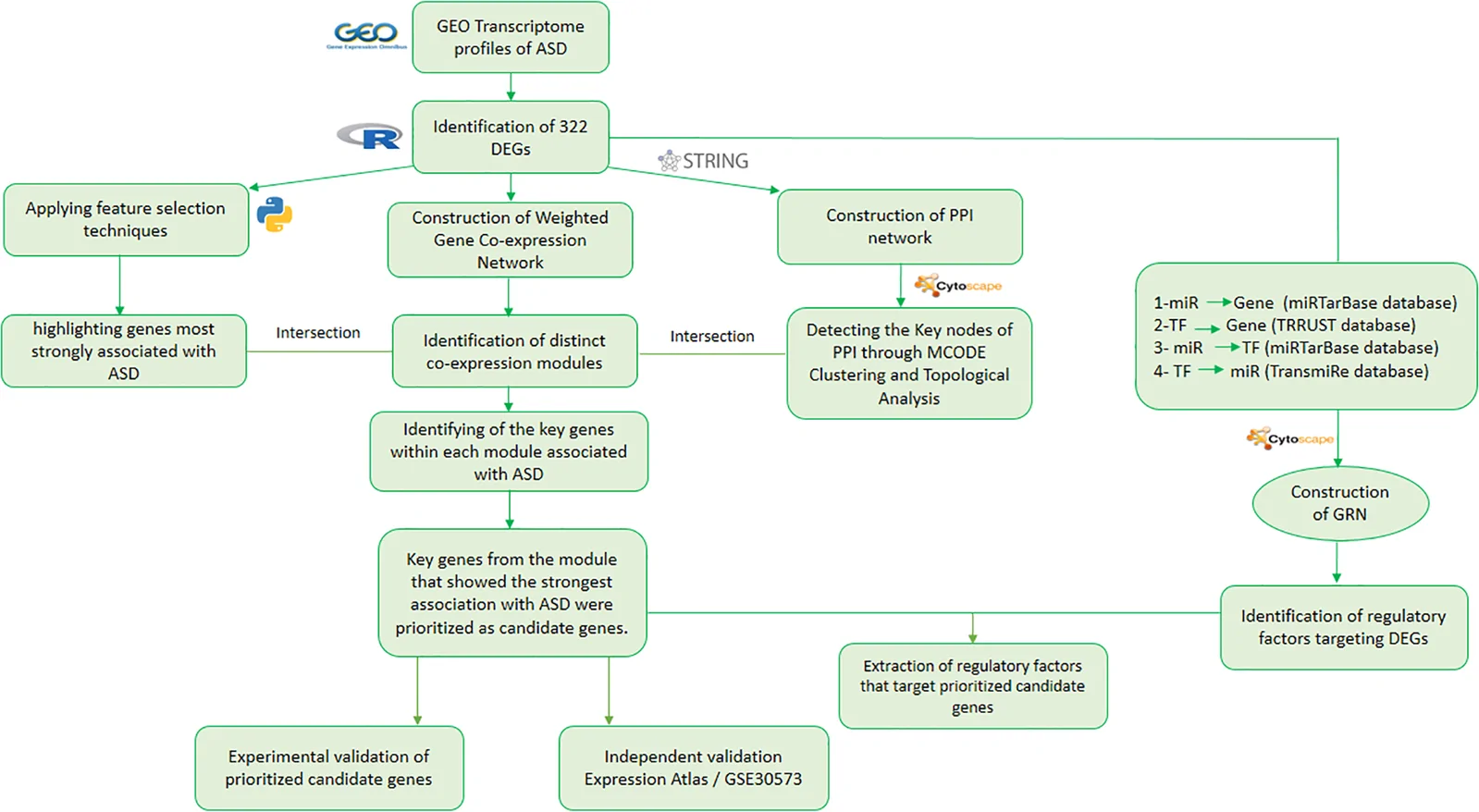
Summary of study workflow.

To identify key genes within each module, we selected those that were additionally supported by PPI network analysis and feature selection in machine learning models. In addition, the GRN was used to identify the regulatory factors associated with DEGs. As an experimental validation step, the expression levels of prioritized candidate genes were examined using RT-qPCR in the hippocampus and peripheral blood in the VPA-induced rat model of autism. An overview of the study design is illustrated in Fig 1.

### 2.2. In *Silico* study

#### 2.2.1. Transcriptomic data preprocessing and differential expression analysis.

Transcriptomic data were retrieved from GEO, a publicly accessible database maintained by the National Center for Biotechnology Information (NCBI) (https://www.ncbi.nlm.nih.gov/geo/). Using the keywords “Homo sapiens,” “Autism spectrum disorder,” and “Peripheral blood,” the GSE18123 dataset was identified. This dataset was generated on the Affymetrix Human Genome U133 Plus 2.0 Array (platform GPL570) and uses microarray technology to assess gene expression in peripheral blood samples from individuals with ASD and age-matched controls, comprising 65 ASD and 32 control samples. Gene expression data were downloaded as raw CEL files (GSE18123_RAW.tar) from GEO and imported into the R statistical environment (version 4.3.0) as AffyBatch objects using the ReadAffy function (affy package, version 1.78.0). Preprocessing was performed with the Robust Multi-array Average (RMA) algorithm (rma function, gcrma package version 2.72.0) [14], which applies model-based background correction, quantile normalization across arrays, and probe-set summarization by median polish, yielding expression values on the log2 scale; therefore, no additional log2 transformation was applied [15]. After RMA normalizition, low-intensity probe sets were filtered out to reduce background noise [16]. The RMA-normalized log2 expression matrix was used for differential expression analysis using the limma package (version 3.56.2) [17]: linear models were fitted using lmFit, group contrasts (ASD vs. control) were specified with makeContrasts and applied via contrasts.fit, and moderated statistics were obtained with eBayes. Probe set identifiers were mapped to gene symbols using the hgu133plus2.db annotation package (version 3.13.0). For the identification of DEGs, probes with a nominal p-value < 0.05 and fold change |log_2_FC|  >  0.5 were retained (S1 Table in S1 File). Although multiple-testing correction (FDR) is a standard statistical approach, we maintained the nominal p-value threshold to ensure the inclusion of a sufficiently large gene set for downstream systems-level analyses and to avoid the premature exclusion of relevant biological signaling pathways.

#### 2.2.2. Weighted gene co-expression network analysis (WGCNA).

We applied the R package “WGCNA” to construct a weighted gene co-expression network based on the identified DEGs. First, pairwise correlations between gene expression profiles were calculated to assess gene co-expression. Using a soft-thresholding approach, these correlations were transformed into a weighted adjacency matrix to emphasize strong gene-gene connections. Next, hierarchical clustering and dynamic tree cutting methods were employed to identify distinct co-expression module groups of genes with similar expression patterns. The minimum module size was set to 10 genes [18]. WGCNA was chosen due to its ability to construct weighted networks and identify robust, biologically meaningful gene modules [19]. The association between each module and ASD was quantified using Pearson correlation between module eigengenes and the ASD trait. Modules with statistically significant correlations (p < 0.05) were selected.

#### 2.2.3. The construction of the PPI network.

Protein–protein interactions play vital roles in various cellular processes [20]. In this study, we constructed a PPI network using DEGs, with interaction data retrieved from the STRING database (https://string-db.org/, version 12.0), which integrates both experimentally validated and computationally predicted protein–protein interactions, providing a comprehensive and robust resource for functional network analysis across a wide range of organisms [21]. The medium confidence threshold in STRING (confidence score = 0.4) was applied. The resulting network was visualized and analyzed in Cytoscape (version 3.9.1). We applied topological analysis to identify hub nodes and used the MCODE plugin (Molecular Complex Detection) to detect densely interconnected clusters within the network.

#### 2.2.4. Construction and analysis of the GRN.

To comprehensively understand the regulatory landscape associated with ASD, we identified four key types of regulatory relationships_miRNA-gene, TF-gene, miRNA-TF, and TF-miRNA_using a curated list of DEGs and established biological databases. The identified DEGs were used as input for the GRN construction, and all regulatory interactions available in the selected databases for these DEGs (and the TFs identified from them) were retrieved and included in the GRN.

**2.2.4.1. miRNAs regulating DEGs**

We utilized the miRTarBase database (http://miRTarBase.mbc.nctu.edu.tw/, miRTarBase 2025), a resource containing millions of experimentally validated miRNA-target interactions, to identify miRNAs that regulate DEGs. miRTarBase integrates data from a wide range of experimental studies and provides robust evidence for miRNA-mRNA interactions [22], making it a reliable tool for uncovering post-transcriptional regulatory mechanisms relevant to ASD.

**2.2.4.2. TFs regulating DEGs**

The TRRUST database (https://www.grnpedia.org/trrust/, v2) was employed to determine which DEGs also function as TF and to map their regulatory relationships with other DEGs. TRRUST compiles transcriptional regulatory interactions in humans and mice, extracted through advanced text mining and manual curation [23], enabling the identification of TF-gene regulatory relationships involved in ASD pathophysiology.

**2.2.4.3. miRNAs regulating TFs**

Regulatory miRNAs targeting the identified TFs were also extracted from miRTarBase, further expanding the network of post-transcriptional regulation and highlighting the interplay between miRNAs and TFs in gene expression control.

**2.2.4.4. TFs regulating miRNAs**

To complete the regulatory network, the TransmiR database (https://www.cuilab.cn/transmir, v3.0) was used to identify TFs that regulate miRNA expression. TransmiR integrates high-throughput ChIP-seq data and extensive manual literature curation, providing detailed information on TF-miRNA regulatory relationships [24].

Finally, all four types of regulatory interactions (miR-gene, TF-gene, miR-TF, and TF-miR) were integrated to construct a GRN. The network was visualized using Cytoscape. Topological analysis was performed with Cytoscape's network analyzer tool, and nodes ranked in the top 10% by degree were extracted as key regulatory elements. Finally, the GRN was used to identify regulatory factors associated with the DEGs.

#### 2.2.5. Machine learning and feature selection.

We applied a multi-step machine learning framework implemented in Python (version 3.12.3) to extract a biologically relevant and robust subset of genes for further analysis. The workflow consisted of data preprocessing, feature selection with hyperparameter optimization, and sensitivity analysis to evaluate the stability of selected features.

**2.2.5.1. Preprocessing**

The GSE18123 dataset comprised 322 DEGs across 97 individuals (65 with ASD and 32 healthy controls). The data were organized with individuals as rows and genes as columns, with a label column indicating class membership (1 for ASD, 0 for control). Feature matrices and label vectors were converted to NumPy (version 1.26.4) arrays prior to model implementation. To enable unbiased performance evaluation, 20% of the samples (20 individuals) were randomly set aside as a test set using stratified sampling implemented through the train_test_split function in scikit-learn (version 1.7.2). The remaining 77 samples formed the training set, which exhibited class imbalance (52 ASD vs. 25 controls). To address this issue, a two-step resampling strategy was employed. First, random under-sampling was applied to slightly reduce the majority class. Subsequently, SMOTE (Synthetic Minority Over-sampling Technique) was used to increase the number of control samples, resulting in a balanced training dataset containing 45 ASD and 45 control samples (90 samples in total). The test set remained untouched throughout model training and feature selection.

**2.2.5.2 LASSO-based feature selection**

Feature selection was first performed using the Least Absolute Shrinkage and Selection Operator (LASSO), which imposes an L1 regularization penalty to shrink less informative gene coefficients to zero, thereby producing a sparse and interpretable model. To avoid arbitrary parameter selection, the regularization parameter (α) was optimized using 5-fold cross-validation within the training set. A logarithmically spaced range of α values (10^-5^ to 10^-3^) was evaluated using the LassoCV implementation in Scikit-learn. The optimal α value was selected based on cross-validated performance, and genes with non-zero coefficients under this optimal model were retained. This procedure resulted in the selection of 66 candidate genes.

**2.2.5.3. Random forest feature importance**

In parallel, a random forest classifier implemented in the scikit-learn library was employed to capture nonlinear effects and gene–gene interactions that may not be detected by linear models. Model hyperparameters, including the number of trees, tree depth, minimum samples for node splitting, and the number of features considered at each split, were optimized using grid search with 5-fold cross-validation. Model selection was guided by the area under the ROC curve (AUC). All 322 genes were ranked according to their random forest importance scores under the optimized model.

**2.2.5.4. Intersection of the two feature selection algorithms and sensitivity analysis**

The final gene sets were obtained by taking the intersection between genes selected by LASSO and the top-ranked genes from the random forest at different importance thresholds. Rather than applying a fixed and arbitrary cutoff, we conducted a sensitivity analysis across multiple random forest importance thresholds. Specifically, intersections were computed between the LASSO-selected genes and the top-ranked random forest genes at several percentile cutoffs (50%, 65%, 80%, and 95% of random forest-ranked features). For each resulting gene subset, a logistic regression classifier was trained with hyperparameter optimization using 5-fold cross-validation on the balanced training set, and model performance was evaluated on the independent test set. The gene subset yielding the most optimal performance across accuracy, precision, recall, F1-score, and AUC was selected as the final feature set for downstream analyses.

#### 2.2.6. Identification of the key genes within each module associated with ASD.

To pinpoint the key genes within each module, we implemented an integrative strategy combining network analysis and a machine learning framework. Specifically, we intersected the genes present in each co-expression module with two criteria: (i) the key nodes of the PPI network and (ii) the genes selected through feature selection techniques. This comprehensive approach enabled us to prioritize genes that are both functionally significant and computationally robust within the ASD molecular landscape. Ultimately, key genes from the module showing the strongest correlation with ASD were prioritized as candidate genes. We used Prism (version 8.4.0) and the t-test to visualize differential expression of key genes in ASD patients compared to the control group.

#### 2.2.7. Independent validation of prioritized candidate genes.

We utilized the Expression Atlas database (https://www.ebi.ac.uk/gxa/home; 2026 update), which serves as a comprehensive resource for exploring gene and protein expression patterns across multiple organisms and diverse disease conditions [25], to provide independent validation of our findings. This resource was employed to assess whether the differential expression patterns of the prioritized candidate genes were consistent with findings reported in independent datasets examining gene expression in patients with ASD.

#### 2.2.8. Functional enrichment analysis.

To elucidate the biological significance of our findings, we performed functional enrichment analysis using the DAVID online platform (https://david.ncifcrf.gov, 2021 update), the database for annotation, visualization, and integrated discovery [26]. Functional enrichment is a widely adopted strategy for uncovering over-represented biological annotations within a specific set of genes, thereby providing insight into the underlying molecular mechanisms [27]. Functional enrichment analysis was performed on three gene sets: (1) identified up-regulated DEGs, (2) genes within the WGCNA module showing the strongest correlation with ASD, and (3) targets of the identified miRNAs (which were predicted to regulate our prioritized candidate genes) that were also present in our DEG list. miRNAs target genes were retrieved from the miRTarBase database. These genes were subjected to Gene Ontology (GO) enrichment analysis to identify significantly enriched biological processes (BP), molecular functions (MF), and cellular components (CC). In addition, pathway enrichment analysis was conducted using Reactome Knowledgebase (https://reactome.org/, version 96) [28] and the Kyoto Encyclopedia of Genes and Genomes (KEGG) database (https://www.kegg.jp/, release 117.0) [29] to identify significantly enriched biological pathways. Enrichment results were considered statistically significant at an FDR < 0.05 for upregulated DEG analyses, and at a nominal p-value < 0.05 for module genes and miRNA targets analyses.

### 2.3. Experimental validation of prioritized candidate genes

#### 2.3.1. Animal care and VPA-induced autism model.

Female and male Wistar rats (200–250 g) were obtained from the animal facility of Shahid Beheshti University of Medical Sciences and maintained under a 12:12 h light–dark cycle at a controlled temperature of 24 ± 2°C, with free access to food and water. Animals were mated overnight, and the presence of spermatozoa in vaginal secretion the following morning was designated as the first day of pregnancy (E1.0). Pregnant females were kept individually and randomly assigned to either the VPA or the control group. Animals in the VPA group received a single intraperitoneal injection of valproic acid (VPA; Sigma-Aldrich, St. Louis, MO, USA) at a dose of 600 mg/kg on embryonic day 12.5 (E12.5) to establish a VPA-induced autism model in the offspring [30]. Control animals received an equivalent volume of physiological saline. All procedures were approved by the Ethics Committee of Shahid Beheshti University of Medical Sciences (Approval code: IR.SBMU.AEC.1403.065) and conducted according to the NIH Guide for the Care and Use of Laboratory Animals.

#### 2.3.2. Postnatal handling and tissue collection.

Following birth, male offspring were selected from the two experimental groups (autism model and control), with four animals per group (n = 4) included in the study. The pups remained with their mothers until postnatal day 21 (P21) and were then weaned and housed individually with free access to food and water until postnatal day 42 (P42). On P42 [30], animals were anesthetized with a combination of xylazine and ketamine. Blood samples were collected via cardiac puncture, and hippocampal tissues were rapidly dissected, snap-frozen in liquid nitrogen, and subsequently stored at –80°C until RNA extraction and qRT-PCR analysis.

#### 2.3.3. RNA extraction and qRT-PCR.

Total RNA was extracted from hippocampal tissues and peripheral blood samples using the Super RNA Extraction Kit (Verner, Aco Teb International, Tehran, Iran) following the manufacturer’s instructions. RNA quality and concentration were assessed using a Nanodrop spectrophotometer (Thermo Fisher Scientific, USA). Complementary DNA (cDNA) was synthesized using the cDNA synthesis kit (Verner, Aco Teb International, Tehran, Iran). qRT-PCR was performed using SYBR Green Master Mix (BioFACT Co., Ltd., Daejeon, South Korea) and gene-specific primers on an Ambion‑Applied Biosystems sequence detection system (USA). Threshold cycles (Ct) were recorded, and relative expression levels of target mRNAs were calculated using the 2^− △△ct^ method, normalized to GAPDH as the housekeeping gene. Each experimental group included four biological samples (n = 4), and reactions were run in duplicate. The primers used for amplification are presented in Table 1.

**Table 1:** qPCR primer sequences.

Gene	Forward primer (5′–3′)	Reverse primer (5′–3′)	Product length (bp)
TLR8	AGGACCACACTGCACTGCTA	GGGACATGTTTTCCCCTTTCC	84
CASP4	CCTGGGGAAGTGTGGATCAG	TGTAGGACAAGTGGTGTGGTG	154
GAPDH	GTGTTCCTACCCCCAATGTGT	ATTGTCATACCAGGAAATGAGCTT	248

## 3. Result

### 3.1. Data pre-processing and identification of DEGs

**Fig 2:**
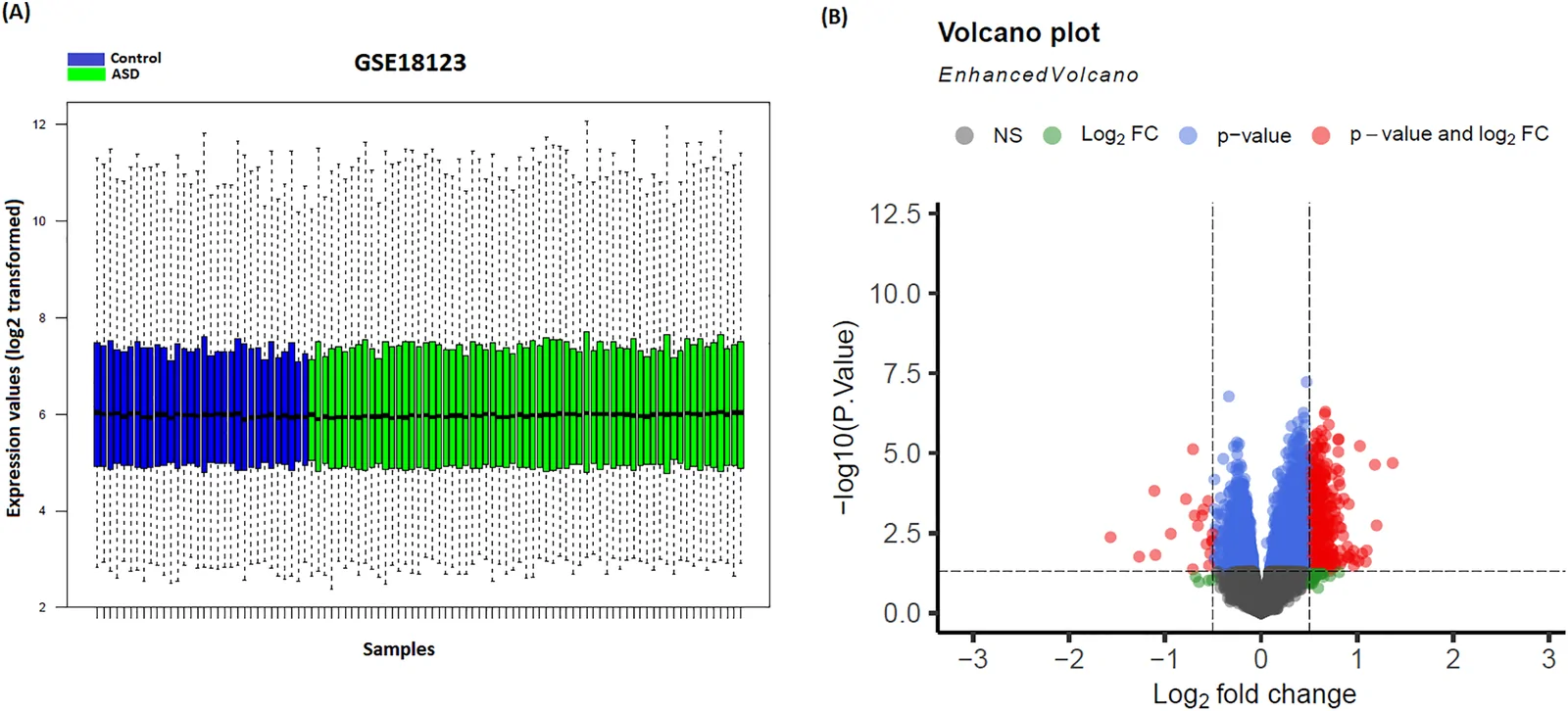
Evaluation of microarray data quality for GSE18123 and detection of DEGs. (A) Boxplot illustrating the distribution of normalized intensity values. (B) Volcano plot depicting DEGs in the peripheral blood samples from individuals with ASD compared to the control group.

The GSE18123 dataset underwent normalization using the limma R package to minimize technical variability, as shown in the box plot of normalized expression data (Fig 2A). Differential expression analysis with limma identified 322 genes (308 up-regulated, 14 down-regulated) in the peripheral blood of ASD patients compared to controls, using thresholds of p-value < 0.05 and |log_2_FC| > 0.5 (S1 Table in S1 File). A volcano plot visualized these DEGs, highlighting their fold changes and statistical significance (Fig 2B). This aligns with methodologies described in other studies analyzing GSE18123, though the exact DEG count varies slightly depending on filtering criteria [31].

### 3.2. Co-expression network construction and hub module identification

**Fig 3:**
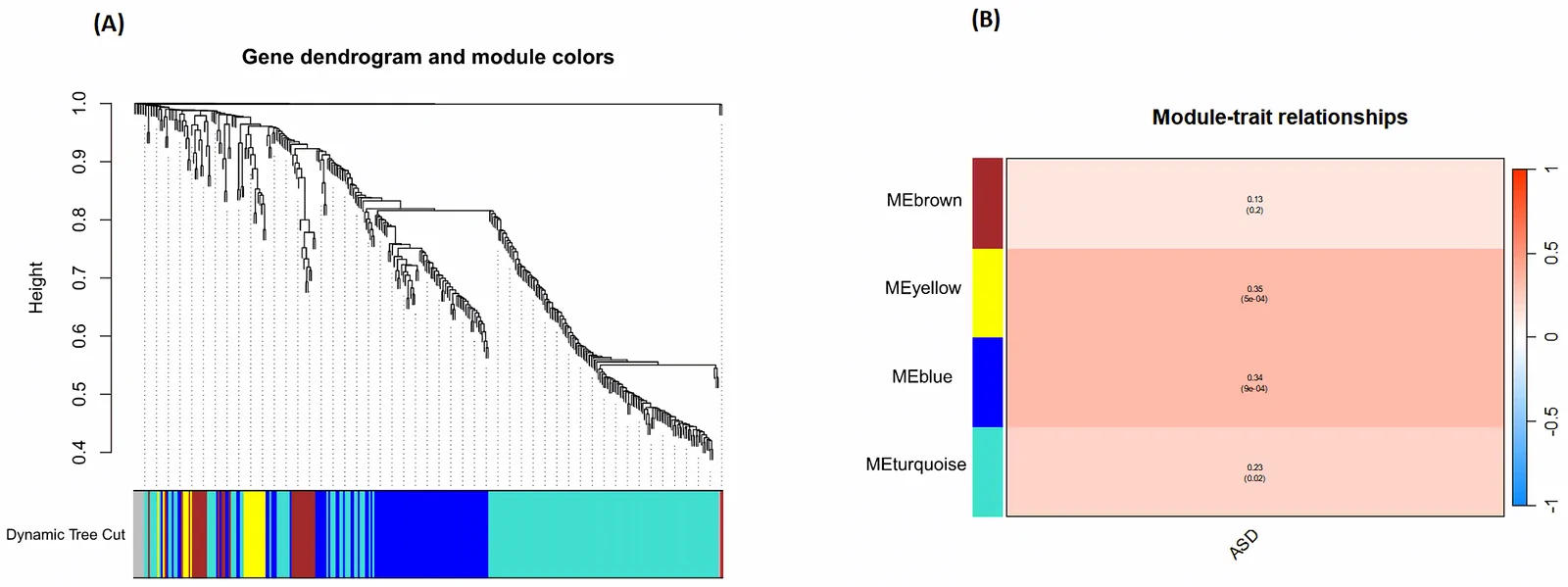
WGCNA of DEGs in the peripheral blood of individuals with ASD compared to the control group. (A) Four co-expression modules (yellow, turquoise, brown, and blue) were detected. (B) Association between gene modules and ASD. The yellow module demonstrates the strongest correlation.

WGCNA was applied to the GSE18123 expression dataset to investigate co-expression patterns among DEGs associated with ASD. The analysis used a soft-thresholding power (β) of 6 to construct a scale-free network, followed by the calculation of the topological overlap matrix (TOM) to cluster genes into modules based on their expression similarity (Fig S1 in S1 File). This approach identified four distinct gene modules: blue (95 genes), brown (30 genes), yellow (18 genes), and turquoise (170 genes) (Fig 3A and S2 Table in S1 File). Among these, the yellow module demonstrated the strongest correlation with ASD status (correlation coefficient = 0.35, p = 0.0005), suggesting its potential relevance to disease mechanisms (Fig 3B). This modular network approach enabled the identification of co-expressed gene clusters potentially involved in ASD-related biological processes.

### 3.3. Construction and analysis of the GRN

The GRN for ASD was constructed by integrating multiple layers of regulatory interactions using curated databases. First, miRNA–mRNA interactions were identified via the MirTarBase database, resulting in 9,163 predicted regulatory relationships between miRNAs and DEGs in the peripheral blood of ASD patients (S3 Table in S1 File). Next, TFs regulating DEGs were determined using the TRUSST database, revealing that 16 of the 322 DEGs functioned as TFs, with 5 regulatory relationships identified between these TFs and DEGs (S4 Table in S1 File). Additionally, MirTarBase was used to uncover 620 miRNA–TF interactions (S5 Table in S1 File), and the TransmiR database provided 644 TF–miRNA regulatory relationships (S6 Table in S1 File).

**Fig 4:**
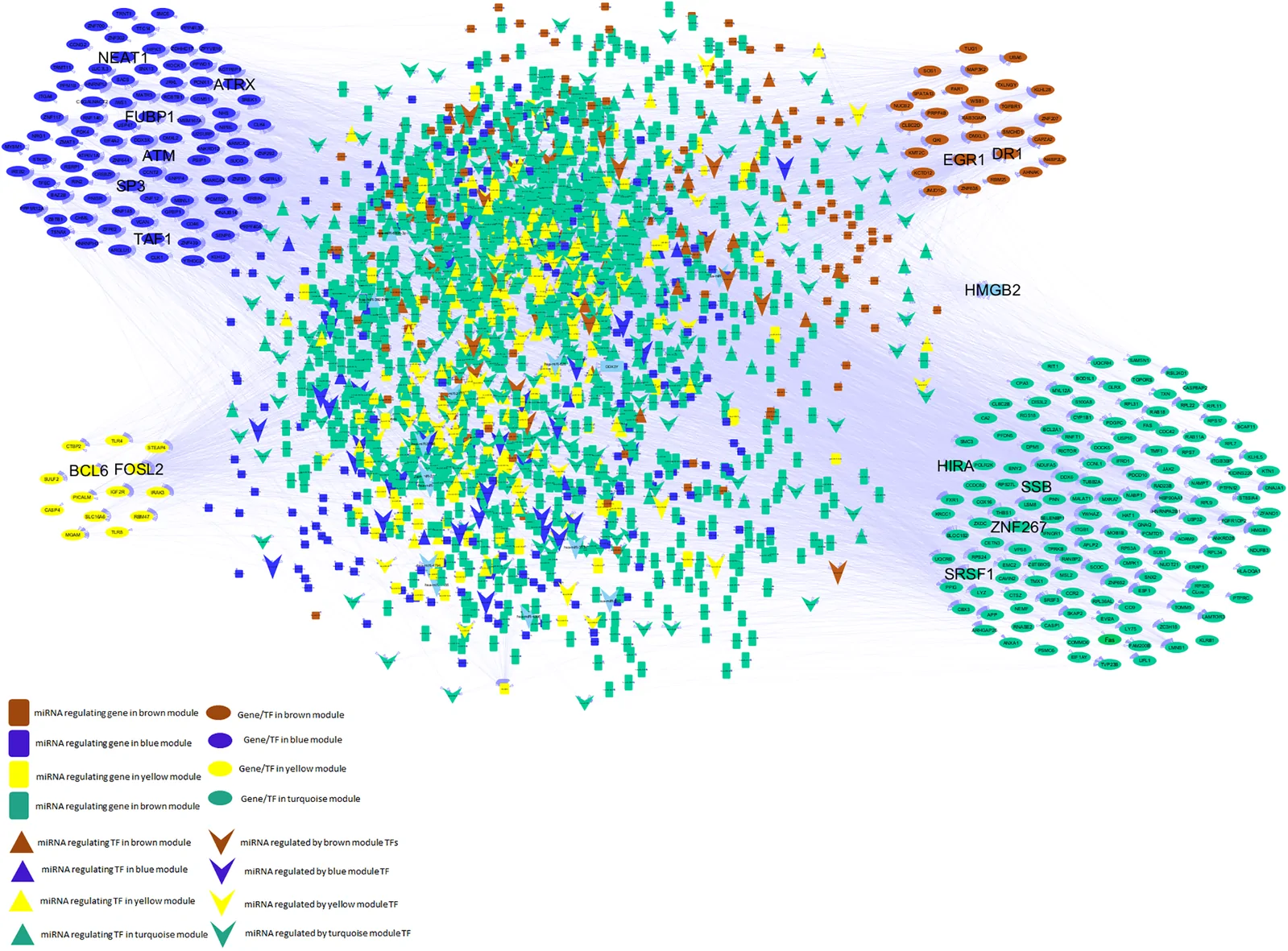
Gene regulatory network. The GRN was constructed based on identified miRNA-gene, TF-gene, miRNA-TF, and TF-miRNA regulatory relationships. Four co-expression modules and their associated regulators were extracted from the GRN. Nodes with larger text size in each module also act as TFs.

The comprehensive GRN, visualized in Cytoscape, integrates miRNA–gene, TF–gene, miRNA–TF, and TF–miRNA interactions, comprising 2,406 nodes and 9,811 edges. Topological analysis identified hub nodes in the top 10% by degree, including key TFs, genes, and miRNAs (S7 Table in S1 File). Finally, the co-expression modules and their associated regulatory factors were extracted from the GRN for further analysis (Fig 4).

### 3.4. Construction and analysis of the PPI network

**Fig 5:**
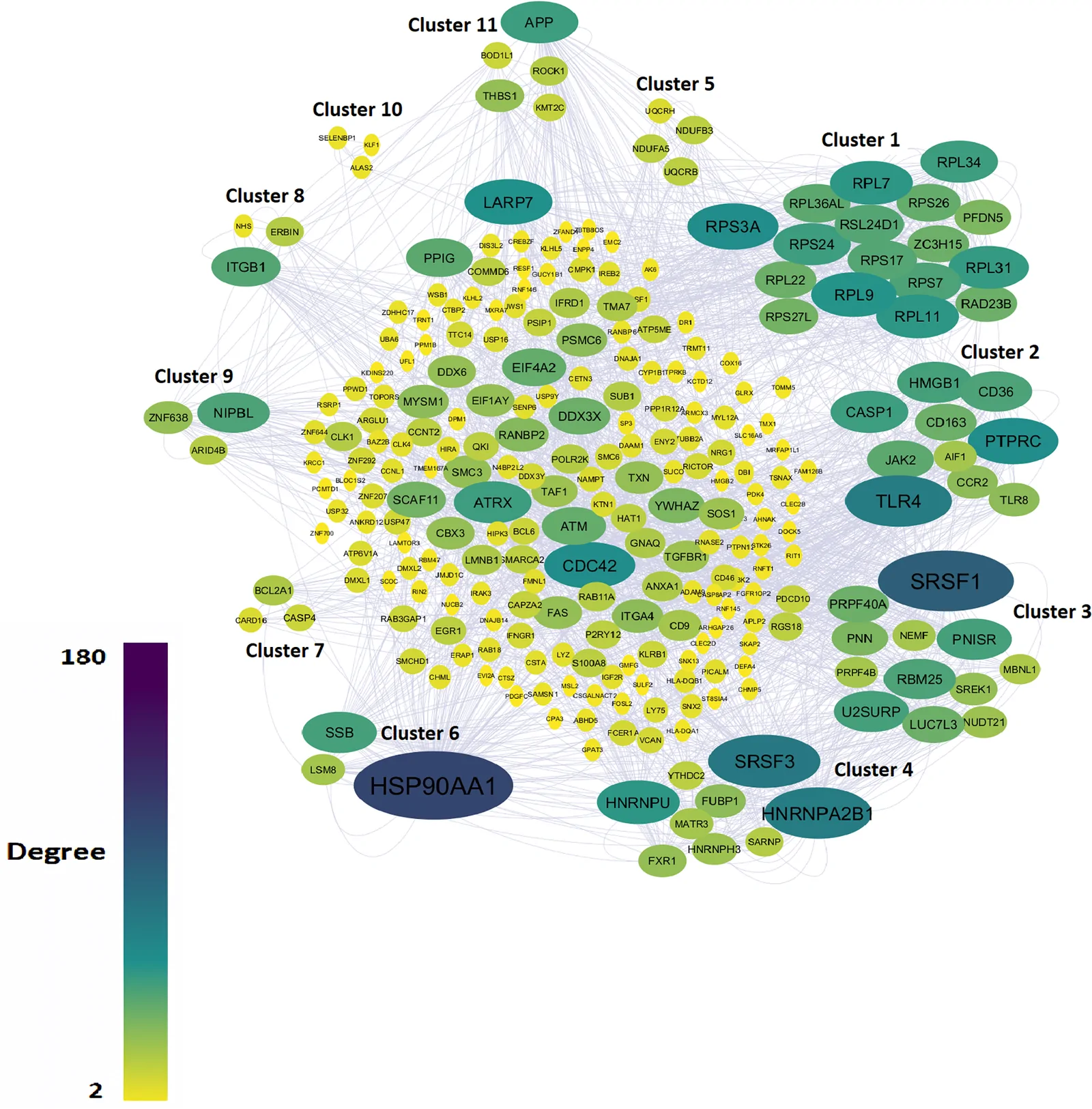
PPI network of DEGs. Eleven clusters were identified by the MCODE algorithm and represented in the PPI network. Node size and color were scaled according to the degree.

The PPI network for the 322 DEGs was constructed and visualized using Cytoscape, resulting in a network comprising 266 nodes and 2,364 edges (Fig 5). The MCODE plugin was employed to identify densely interconnected regions within this network, revealing 11 distinct clusters. All detected clusters (MCODE score ≥ 2.5) were subjected to subsequent analysis (S8 Table in S1 File).

Topological analysis was performed the nodes exhibiting a degree ≥ 15 were identified as hub nodes (S9 Table in S1 File). The hub nodes and the genes within the MCODE clusters were merged to highlight key nodes in the PPI network (S10 Table in S1 File). This combined analysis provided insights into the modular organization of the PPI network and highlighted both topologically important hub genes and potential functional complexes.

### 3.5. Machine learning and feature selection

Following preprocessing, the training dataset was balanced to include 90 samples (45 ASD and 45 controls), while the independent test set remained unchanged. LASSO feature selection with cross-validated regularization identified 66 genes with non-zero coefficients. In parallel, the optimized random forest model ranked all 322 genes according to their importance scores. Consensus feature selection was performed by intersecting LASSO-selected genes with random forest–ranked genes under multiple importance thresholds. Sensitivity analysis demonstrated that model performance on the independent test set varied across thresholds, indicating that feature selection was sensitive to the number of retained genes (Table 2). The subset containing 63 genes (S11 Table in S1 File) achieved the highest classification performance across most metrics. The confusion matrix for the best-performing 63-gene model on the independent test set is provided (Table 3).

**Table 2:** Logistic regression performance across consensus gene set sizes. Performance of logistic regression on the independent test set using consensus gene sets derived from the intersection of LASSO and random forest feature selection results.

Number of Genes	accuracy	precision	recall	F1-score	Auc
37	0.90	1.000	0.846	0.917	0.978
47	0.85	0.917	0.846	0.880	0.978
58	0.85	0.917	0.846	0.880	0.978
63	0.90	0.923	0.923	0.923	0.978

**Table 3:** Confusion matrix of the predictive model performance. Classification results of the ASD group versus controls.

Actual \ Predicted	ASD	Control
ASD	12	1
Control	1	6

### 3.6. Identification of the key genes within each module associated with ASD

**Fig 6:**
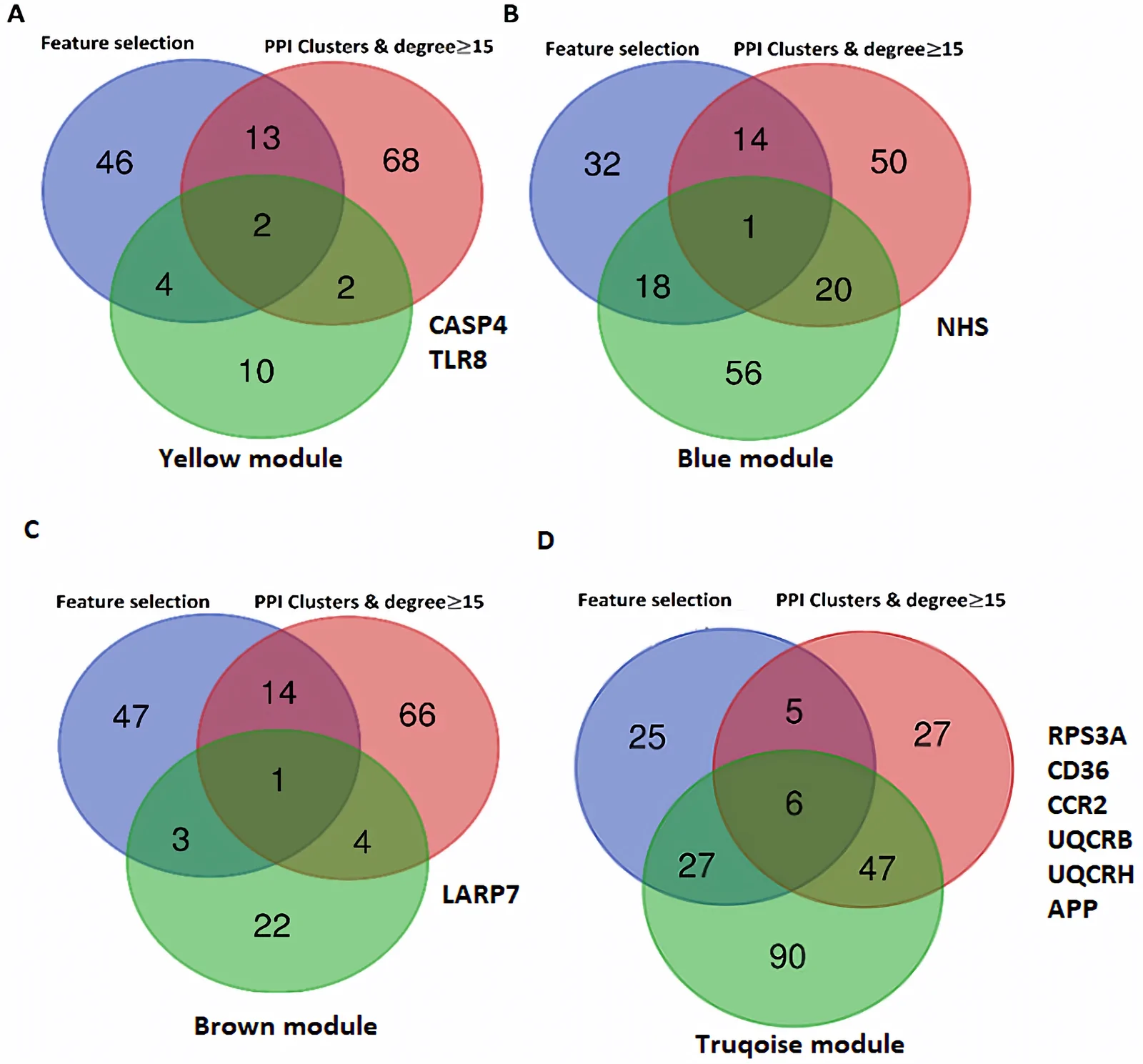
Intersection of genes of each module with key nodes of the PPI network and genes from feature selection.

**Fig 7:**
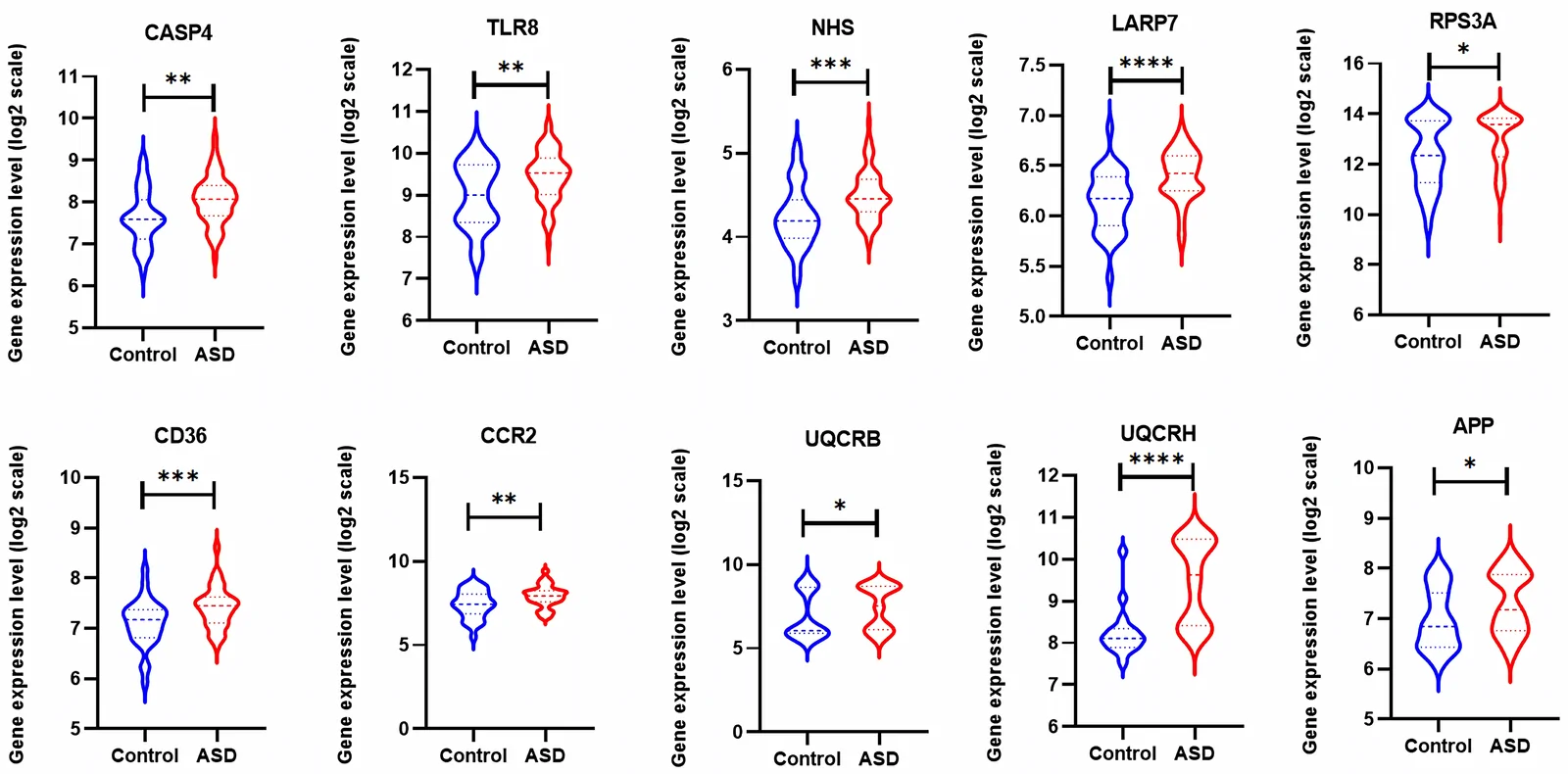
Differential expression of key genes from co-expression modules associated with ASD. Violin plots show log2-normalized gene expression levels in ASD patients and the control group. Statistical significance was determined using t-test (*P < 0.05, **P < 0.01, ***P < 0.001, ****P < 0.0001).

To systematically identify key genes within each module, we integrated the results obtained from co-expression network analysis, PPI network analysis, and feature selection techniques. Specifically, for each co-expression module, key genes were identified by intersecting the module genes with key nodes of the PPI network and the genes selected through feature selection. Using this stringent criterion, we identified the following overlapping genes: *TLR8* and *CASP4* (yellow module), *NHS* (blue module), *LARP7* (brown module), and *RPS3A*, *CD36*, *CCR2*, *UQCRB*, *UQCRH*, and *APP* (turquoise module) (Fig 6 and S12 Table in S1 File). The changes in the expression level of these genes in the ASD patients compared to the control group are shown in Fig 7. All identified key genes were upregulated in ASD patients compared to the control group.

Among the identified key genes, *TLR8* and *CASP4*, which were located in the module showing the strongest correlation with ASD (yellow module), were prioritized as candidate genes associated with ASD. These genes were not only situated within a co-expression module that was the most relevant to ASD, but also were identified as key nodes of the PPI network and selected by feature selection.

### 3.7. Construction of a GRN Sub-network containing yellow module genes

**Fig 8:**
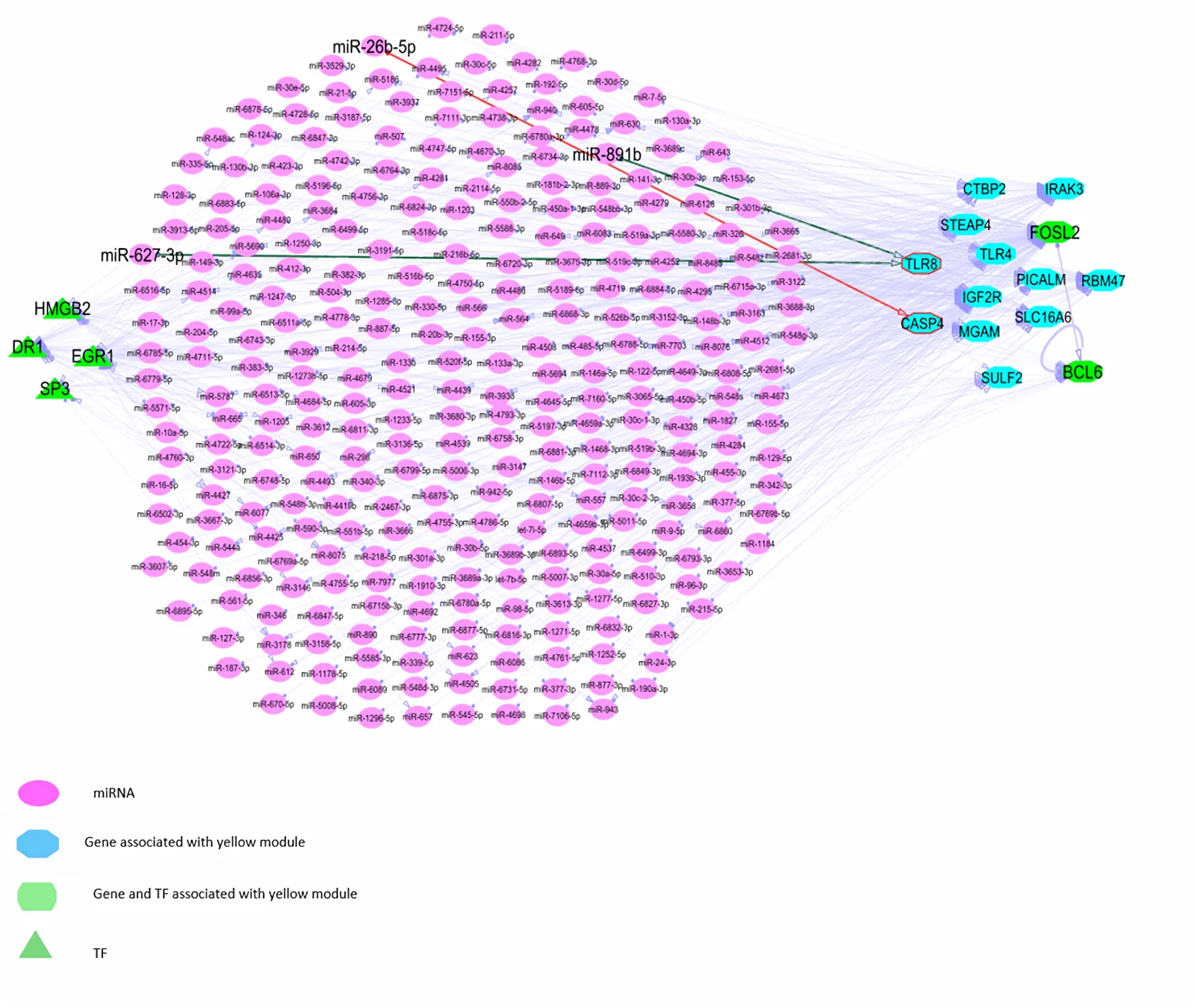
The GRN sub-network. This network, containing yellow module genes, shows interactions between module genes and their regulatory factors.

To further elucidate the regulatory landscape of genes within the yellow module, which showed the strongest correlation with ASD, we extracted a sub-network from the GRN containing yellow module genes and their regulatory relationships, including miRNA–gene, TF–gene, miRNA–TF, and TF–miRNA interactions (Fig 8 and S13 Table in S1 File). *TLR8* and *CASP4*, which were located within the yellow module and had been prioritized as candidate genes associated with ASD, are predicted to be regulated at the post-transcriptional level by the identified miRNAs, including miR-891b and miR-627-3p for *TLR8* and miR-26b-5p for *CASP4*.

### 3.8. Independent validation of prioritized candidate genes

Independent validation results from the Expression Atlas database showed that, consistent with our findings, *CASP4* and *TLR8* were significantly upregulated in the GSE30573 dataset. This dataset analyzed post-mortem brain tissue samples from individuals with ASD compared with neurotypical controls [32]. Specifically, both genes exhibited significant differential expression (*CASP4*: log_2_ FC = 2.0, adjusted p-value = 1.47 × 10^-16^; *TLR8*: log_2_ FC = 2.2, adjusted p-value = 0.00126). These results further support the potential relevance of *CASP4* and *TLR8* to ASD.

### 3.9. Functional enrichment analysis

#### 3.9.1. Functional enrichment analysis of the DEGs.

**Fig 9:**
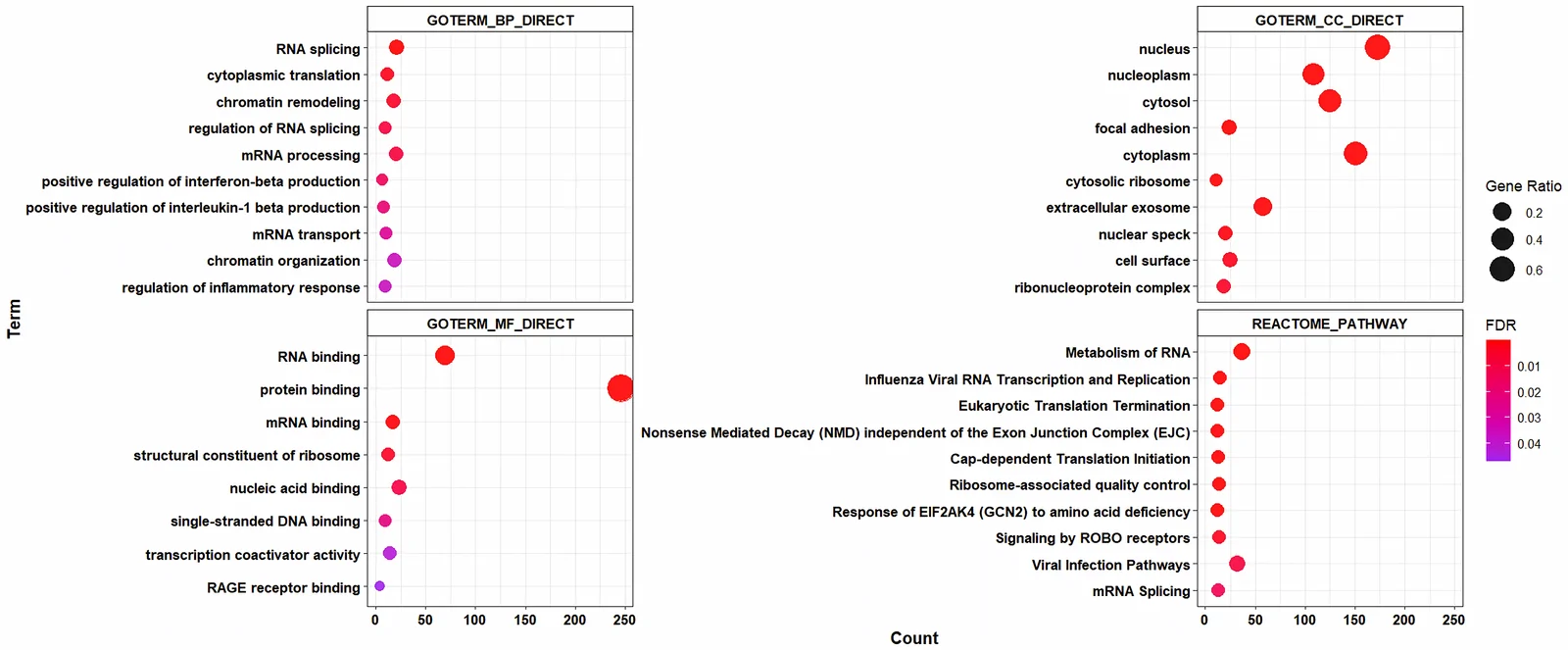
GO and KEGG pathway enrichment analysis of upregulated DEGs. For each category, up to 10 selected significantly enriched terms are presented as dot plots (FDR < 0.05).

The 308 upregulated DEGs (S14 Table in S1 File) were subjected to GO and Reactome enrichment analysis. Downregulated genes did not show any significant results and were therefore not reported. The enrichment analysis of the upregulated DEGs revealed that the terms showing significant enrichment in the Biological Process (BP) were mostly associated with RNA splicing, cytoplasmic translation, chromatin remodeling, mRNA processing, regulation of RNA splicing, positive regulation of interferon-beta production, positive regulation of interleukin-1 beta production, mRNA transport, chromatin organization, and regulation of the inflammatory response. The significantly enriched MF terms were mainly associated with RNA binding, protein binding, mRNA binding, structural constituent of ribosome, nucleic acid binding, single-stranded DNA binding, transcription coactivator activity and RAGE receptor binding. Regarding the cellular components CC, the enriched items were mainly focused on the nucleus, nucleoplasm, cytosol, focal adhesion, cytoplasm, cytosolic ribosome, extracellular exosome, nuclear speck, cell surface, ribonucleoprotein complex, cytoplasmic stress granule, ribosome, cytosolic large ribosomal subunit, NLRP3 inflammasome complex, cytosolic small ribosomal subunit, PML body, canonical inflammasome complex and lamellipodium. Reactome pathway analysis indicated the up-regulated DEGs are significantly involved in pathways related to the metabolism of RNA, influenza viral RNA transcription and replication, eukaryotic translation termination, nonsense mediated decay (NMD) independent of the exon junction complex (EJC), Cap-dependent translation initiation, ribosome-associated quality control, response of EIF2AK4 (GCN2) to amino acid deficiency, signaling by ROBO receptors, viral infection pathways and mRNA splicing. The results of the GO and Reactome analyses are shown in Fig 9 and S15 Table in S1 File.

#### 3.9.2. Functional enrichment analysis of the yellow module genes.

**Fig 10:**
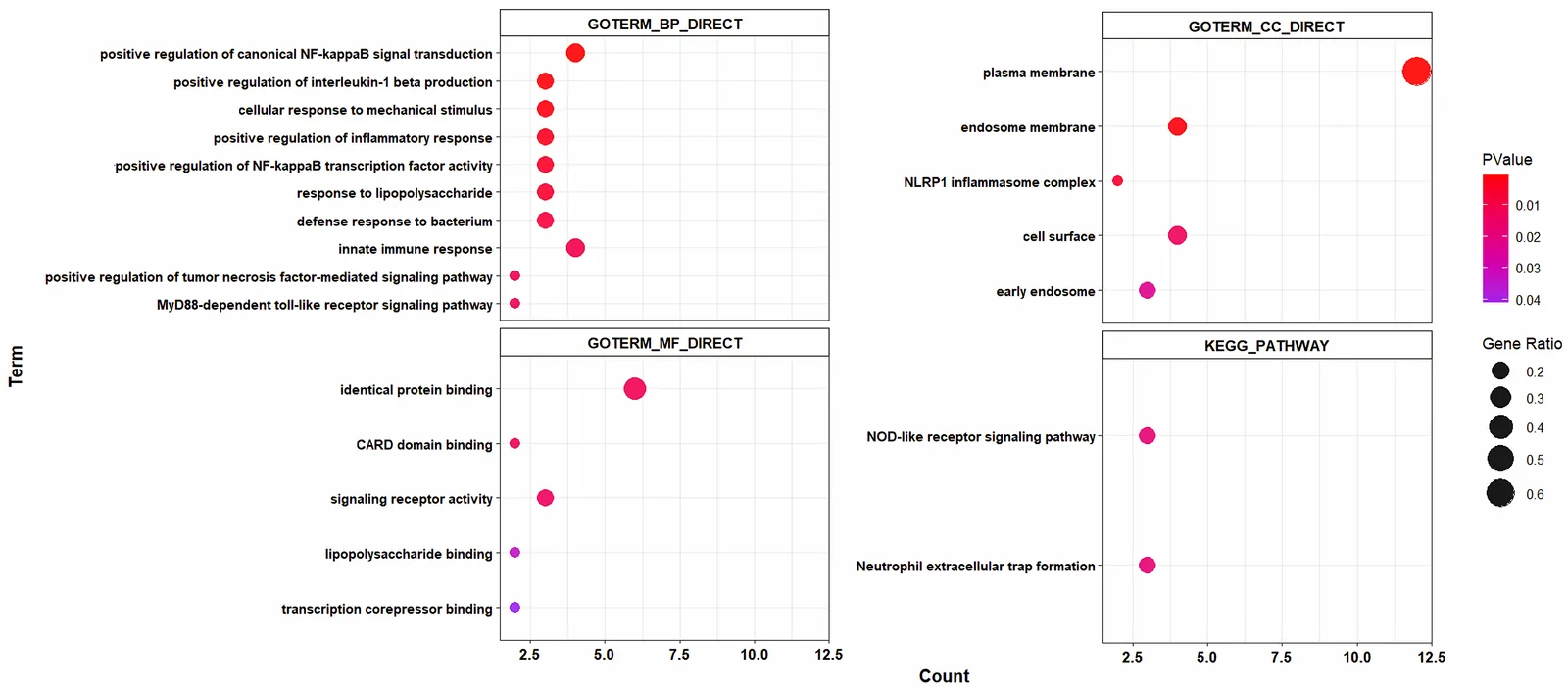
GO and KEGG pathway enrichment of the yellow module genes. For each category, up to 10 significantly enriched terms are presented as dot plots (p-value < 0.05).

We also performed GO and KEGG enrichment analysis for 18 genes in the yellow module (these genes are presented in S2 Table in S1 File). The terms that showed significant enrichment in BP were mostly associated with positive regulation of canonical NF-κB signal transduction, positive regulation of interleukin-1 beta production, cellular response to mechanical stimulus, positive regulation of inflammatory response, positive regulation of NF-κB transcription factor activity, response to lipopolysaccharide, defense response to bacterium, innate immune response, positive regulation of tumor necrosis factor-mediated signaling pathway and MyD88-dependent toll-like receptor signaling pathway. The significantly enriched MF terms were mainly associated with identical protein binding, CARD domain binding, signaling receptor activity, lipopolysaccharide binding, and transcription corepressor binding. In terms of CC, enriched items are the plasma membrane, endosome membrane, NLRP1 inflammasome complex, cell surface, and early endosome. The KEGG pathway analysis showed that the genes of the yellow module are significantly enriched in the NOD-like receptor signaling pathway and Neutrophil extracellular trap formation. The results of the GO and KEGG analyses are shown in Fig 10 and S16 Table in S1 File.

#### 3.9.3. Functional enrichment analysis of miRNAs regulating *CASP4* and *TLR8.*

**Fig 11:**
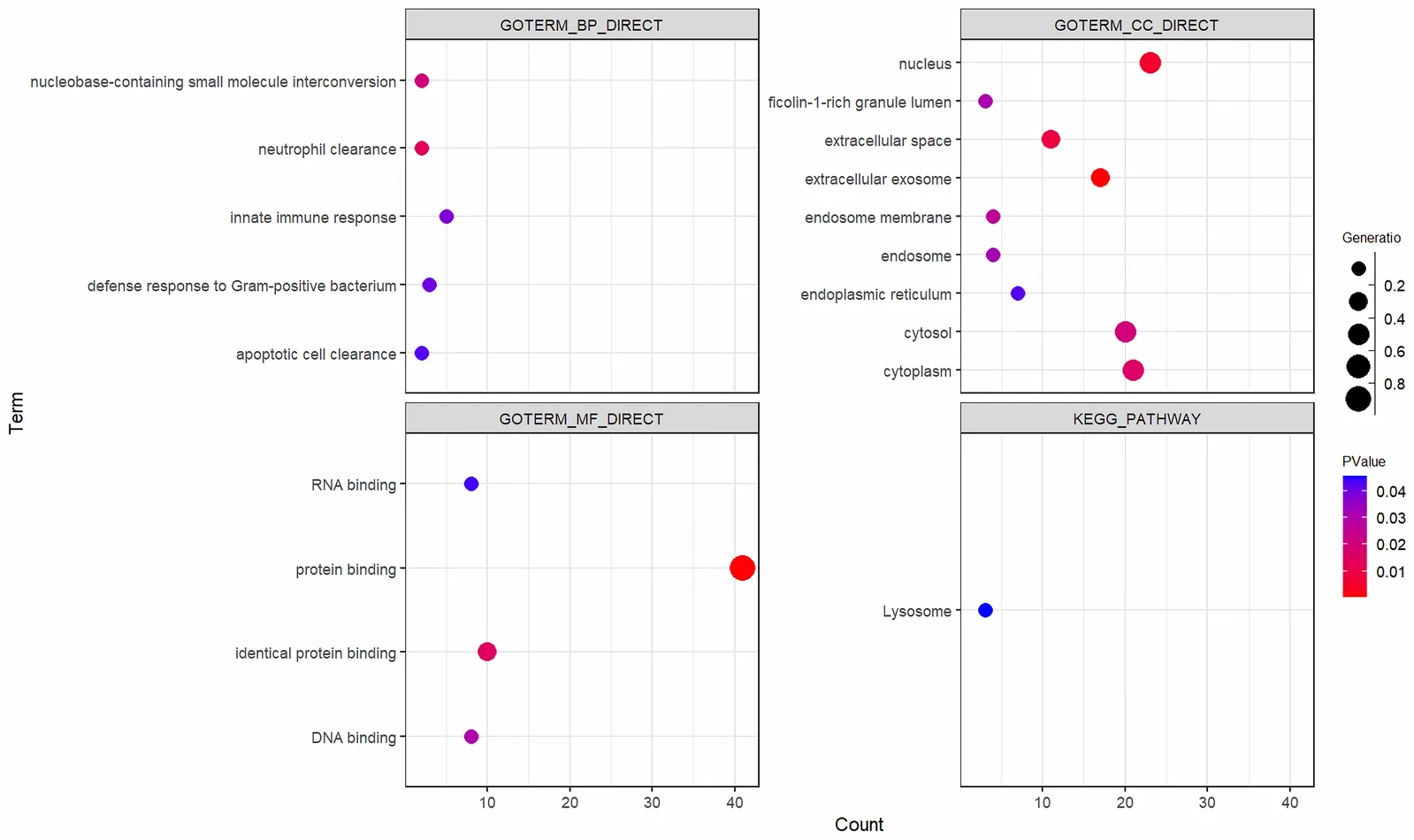
GO and KEGG pathway enrichment analysis of miR-26b-5p target genes. (p-value < 0.05).

**Fig 12:**
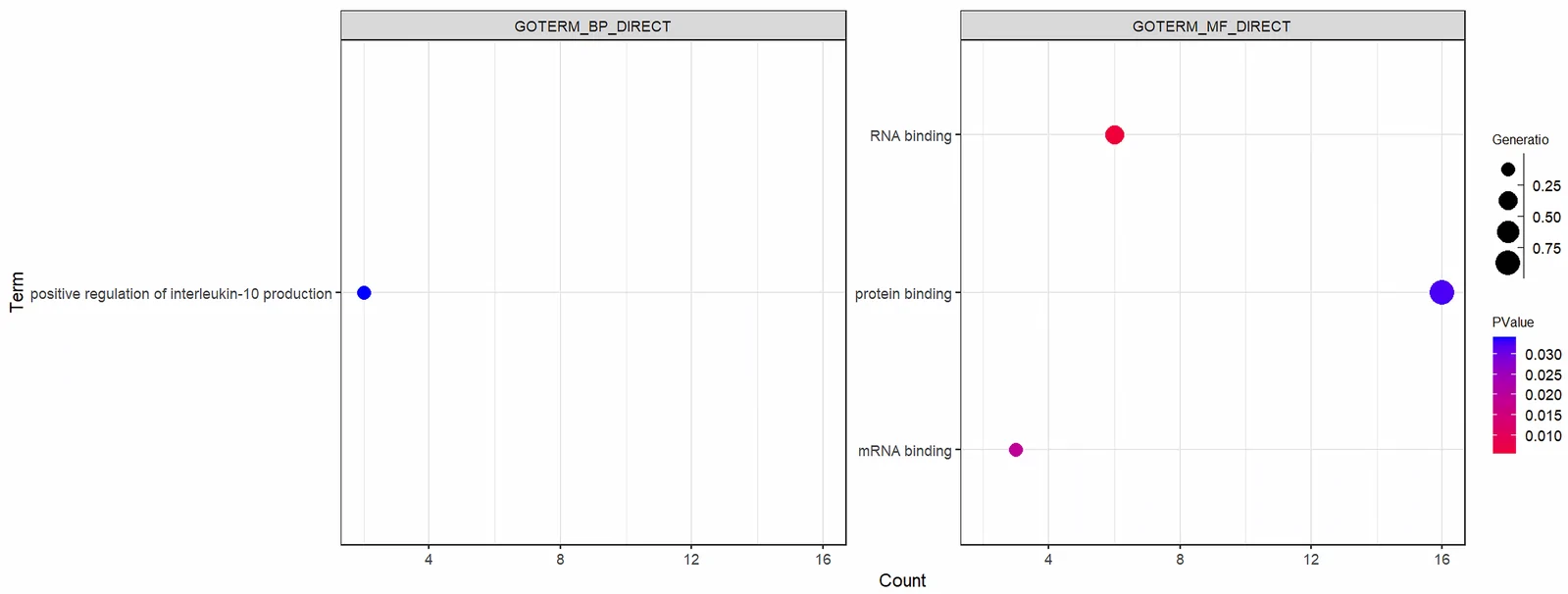
GO enrichment analysis of miR-891b and miR-627-3p target genes. (p-value < 0.05).

miR-26b-5p target genes are significantly enriched in BP related to neutrophil clearance, nucleobase-containing small molecule interconversion, innate immune response, defense response to Gram-positive bacterium, and apoptotic cell clearance. miR-26b-5p target genes in terms of MF were most associated with protein binding, identical protein binding, DNA binding, and RNA binding. The KEGG pathway analysis revealed that miR-26b-5p target genes are significantly enriched in the lysosome. miR-891b and miR-627-3p target genes are significantly enriched in BP related to positive regulation of interleukin-10 production, and in terms of MF were most associated with RNA binding, mRNA binding, and protein binding. The results of the GO and KEGG enrichment analyses for miR-26b-5p target genes are presented in Fig 11 and Table S17 in S1 File, and those for miR-891b and miR-627-3p target genes are presented in Fig 12 and Table S18 in S1 File.

### 3.10. mRNA expression levels of *CASP4* and *TLR8* in a VPA-induced rat model of autism

**Fig 13:**
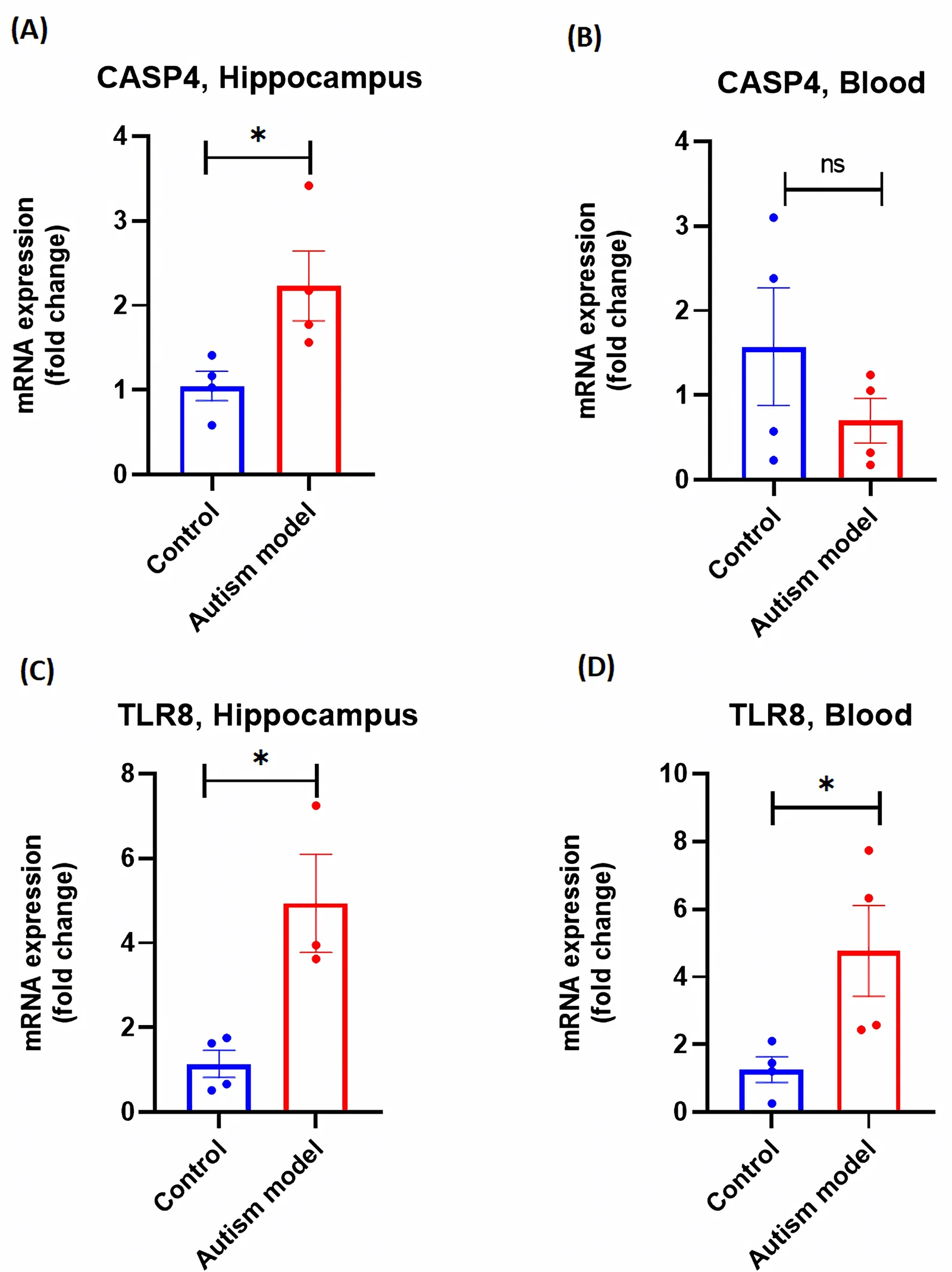
*CASP4* and *TLR8* relative mRNA expression levels in the hippocampus and peripheral blood of control and autism model rats, as measured by qRT-PCR. *CASP4* and *TLR8* were significantly up-regulated in the hippocampus. In peripheral blood, *TLR8* expression was significantly increased, whereas *CASP4* showed no significant change. Data are presented as mean ± SEM (n = 3–4 per group), and statistical significance was assessed using t-test (*P < 0.05).

To validate our in silico predictions, we measured hippocampal and peripheral blood mRNA levels of *CASP4* and *TLR8* in control and VPA-induced autism model rats using qRT-PCR. In the hippocampus, *CASP4* expression was significantly increased in the autism model (p = 0.0387; Fig 13A). Likewise, *TLR8* expression was also significantly up-regulated in the hippocampus (p = 0.0148; Fig 13C). In peripheral blood, *CASP4* expression did not differ significantly between the groups (p = 0.2844; Fig 13B), while *TLR8* expression was significantly increased in the autism model (p = 0.0452; Fig 13D).

## 4. Discussion

The results obtained from the in silico part of this study prioritized *CASP4* and *TLR8* as candidate genes associated with ASD that were upregulated in the peripheral blood of ASD patients. In addition, in another part of this study, experimental validation in a rat model of autism demonstrated increased *TLR8* expression level in both the hippocampus and peripheral blood, whereas *CASP4* expression was elevated specifically in the hippocampus. Another finding of our study, derived from GRN analysis, revealed that miR-891b and miR-627-3p act as regulators of *TLR8*, whereas miR-26b-5p targets *CASP4*.

In recent years, the application of systems biology approaches to elucidate the biological mechanisms underlying complex disorders such as ASD has grown substantially. For example, Zhou et al. (2023) aimed to identify potential novel biomarkers for ASD by using PPI network analysis and constructing a miRNA–mRNA regulatory network [33]. Furthermore, Wei et al. (2022) sought to identify hub genes in ASD by constructing co-expression modules from blood transcriptome data [34]. In this regard, the present study adopted a broader perspective by integrating systems biology and machine learning approaches, which led to the identification of *TLR8* and *CASP4*, and experimental validation confirmed these findings in a rat model of autism.

The Toll-like receptor (TLR) family, as key components of the innate immune system, stimulates immune responses and activates transcription factors that regulate the expression of cytokines, chemokines, growth factors, and other inflammatory mediators [35]. Previous studies indicate that TLR signaling contributes to immune dysregulation and altered inflammatory responses in ASD. For instance, Enstrom et al. (2010) showed that monocyte-derived cells from children with ASD exhibit altered cytokine responses to TLR stimulation, with significantly elevated levels of IL-1β, IL-6, and TNF-α [36]. In addition, increased *TLR4* expression has been reported in peripheral blood immune cells of individuals with ASD [37]. Collectively, these findings suggest that dysregulation of TLR signaling plays an important role in modulating inflammatory mediators and, consequently, may contribute to immune dysfunction in ASD. *TLR8* was identified in the present study as a candidate gene associated with ASD. To date, there is no direct evidence from previous studies implicating *TLR8* in ASD. Given previous research on the association of other TLRs, such as *TLR2*, *TLR3*, and *TLR4*, with ASD [38], and the established role of TLR-dependent signaling pathways in neuroinflammation [39], *TLR8* may represent a promising candidate for further studies on ASD pathophysiology. Furthermore, *TLR8* has been shown to play a functional role in the central nervous system. It is prominently expressed in embryonic axons from embryonic day 12 through the end of the third postnatal week, when the formation of neurons and axons is almost completed, and its expression is downregulated in the mature brain [40]. *TLR8* inhibits neurite outgrowth and promotes neuronal apoptosis through an NF-κB-independent mechanism [41]. It also contributes to dendritic pruning via MYD88-dependent signaling, particularly during late developmental stages [42]. These observations suggest that, in addition to its immune functions, *TLR8* may contribute to ASD by influencing neurodevelopmental processes, including synaptic pruning and neuronal apoptosis, during critical periods of brain development. Given that, in this study, *TLR8* upregulation was confirmed in both hippocampal tissue and peripheral blood in the rat model of autism, this molecule holds potential for future investigation both as a non-invasive diagnostic biomarker and as a molecule functionally associated with ASD pathophysiology.

*CASP4* is another candidate gene associated with ASD identified in this study. It is involved in both inflammatory processes and apoptosis of neuronal and glial cells [43]. *CASP4* plays a key role in activating the non-canonical inflammasome, leading to pyroptosis-induced cell death and amplification of inflammatory responses [44]. Localized to the endoplasmic reticulum (ER) membrane, *CASP4* is cleaved in response to ER stress–inducing stimuli, acting as a pivotal mediator of pyroptosis [45,46]. Recent evidence suggests that pyroptosis is involved in the pathological mechanisms of ASD [47]. Consistent with these results, our study demonstrated increased *CASP4* expression in the hippocampus of the rat model of autism. Notably, *CASP4* has been reported to participate in TNF-α–induced NF-κB activation independently of its catalytic activity, functioning as a scaffolding protein within the TNFR complex. In addition, *CASP4* is required for activation of the IκB kinase complex, a central regulator of NF-κB signaling [48]. Given previous studies supporting a role for NF-κB in ASD etiology [49], this hypothesis is proposed that *CASP4* may act as a modulator of NF-κB signaling, thereby contributing to ASD-related pathological processes. Consistent with these findings, our study suggests that *CASP4* may act as a molecular link between neuroinflammatory processes and neurodevelopmental abnormalities associated with ASD.

We also investigated the GRN to gain further insight into the post-transcriptional regulatory mechanisms involved in the dysregulation of *TLR8* and *CASP4*. Our results indicated that miR-891b and miR-627-3p potentially regulate *TLR8*, whereas miR-26b-5p targets *CASP4*. miRNAs regulate numerous genes and influence entire gene networks that contribute to the pathophysiology of neurodevelopmental disorders, including ASD [50]. Given their roles in neural plasticity [51] and neural development, numerous studies in recent years have examined miRNA expression changes in ASD patients [52], and they have also been investigated as potential biomarkers in various biological fluids and tissues, including serum, blood plasma, saliva, and brain tissue [53]. Although there is no information regarding miR-627-3p, miR-891b has been detected in neurons differentiated from iPSC lines generated from patients with fragile X syndrome [54]. Moreover, miR-26b-5p has been proposed as a potential biomarker in ADHD [55] and Alzheimer’s disease [56], and it has been reported to be associated with the regulation of cell cycle and apoptosis in cancer cells [57,58]. Considering the regulatory effects of these miRNAs on *TLR8* and *CASP4*, two genes exhibiting dysregulated expression in ASD models, and their involvement in multiple diseases, including neurological disorders, these miRNAs may represent promising candidates for further investigation in ASD.

## 5. Limitations and future directions

Although this study combined comprehensives system biology approach with experimental validation in a rat model of autism, several limitations should be acknowledged. Given the network-based and multi-layered nature of our study, we utilized nominal p-values for identifying DEGs to ensure the inclusion of a sufficient gene set for downstream analyses and to avoid the premature exclusion of potentially relevant biological pathways. While this approach increased the risk of Type I errors, we recommend that future studies integrate GEO datasets and apply FDR to ensure statistical robustness and reproducibility of these findings. Future research should investigate the potential of these biomolecules as diagnostic biomarkers and therapeutic targets in these patients. In addition, further experimental studies are required to elucidate the association of the identified biomolecules with ASD, such as gene manipulation in in vitro and in vivo models and validation of the miRNA_mRNA regulatory axis.

## 6. Conclusion

Using integrative in silico analyses and experimental validation, this study suggests *TLR8* and *CASP4*, along with their potential regulatory miRNAs (miR-891b, miR-627-3p, and miR-26b-5p), as candidate biomolecules that may be associated with ASD. *CASP4* and *TLR8* may be involved in ASD pathophysiology through dysregulation of inflammatory signaling, pyroptosis, and synaptic pruning, and can be suggested as potential biomarkers and therapeutic targets for further investigation in these patients.

## Supporting information

S1 File Fig S1 presents the WGCNA analysis of DEGs in the peripheral blood of individuals with ASD compared to the control group. Table S1 presents the differentially expressed genes in the peripheral blood of patients with ASD compared to the control group. Table S2 lists the genes within each detected co-expression module. Tables S3, S4, S5, and S6 contain the four regulatory relationships: miRNA-gene, TF-gene, miRNA-TF, and TF-miRNA. Table S7 shows the hub nodes of the GRN. Tables S8, S9, and S10 highlight the key nodes of the PPI network. Table S11 shows the genes selected using the feature selection method. Table S12 shows the key genes within each module associated with ASD. Table S13 contains the regulatory factors related to the genes involved in the yellow module. Table S14 contains background gene list used for functional enrichment analysis. Table S15 contains the KEGG and GO enrichment analysis of the DEGs. Table S16 contains the KEGG and GO enrichment analysis of the yellow module genes. Tables S17 and S18 present the KEGG and GO enrichment results for the target genes of the identified miRNAs. Table S19 contains RMA-normalized expression matrix of DEGs across GSM samples.
(RAR)
